# PFAS Toxicity: What’s True, What’s Not, and What Really Matters

**DOI:** 10.1021/acs.est.6c01037

**Published:** 2026-05-18

**Authors:** Jamie C. DeWitt, Ian T. Cousins, Gretta Goldenman, Dorte Herzke, Rainer Lohmann, Mark Miller, Carla A. Ng, Martin Scheringer, Xenia Trier, Zhanyun Wang

**Affiliations:** † Department of Environmental Science, 7675 Stockholm University, Stockholm, SE 10691, Sweden; ‡ Milieu Consulting SPRL, Brussels 1060, Belgium; § NILU, Tromsø 9296, Norway and Division of Environmental Health, Norwegian Institute of Public Health, Oslo 0213, Norway; ∥ Graduate School of Oceanography, University of Rhode Island, Narragansett Rhode Island 02882, United States; ⊥ National Center for Advancing Translational Sciences, U.S. Public Health Service, Rockville Maryland 20850, United States; # Department of Civil & Environmental Engineering and Environmental and Occupational Health, 6614 University of Pittsburgh, Pittsburgh Pennsylvania 15261, United States; ¶ Institute of Biogeochemistry and Pollutant Dynamics, ETH Zürich, Zürich, CH 8092, Switzerland; ∇ Department of Plant and Environmental Sciences, Section for Environmental Chemistry and Physics, 4321 University of Copenhagen, Copenhagen 1165, Denmark; ○ Empa-Swiss Federal Laboratories for Materials Science and Technology, St., St. Gallen, CH 9014, Switzerland; ⧫ University of Southern Denmark, Department of Green Technology & Danish Institute for Advanced Study (DIAS), 5230 Odense, Denmark; †† National Centre of Competence in Research (NCCR) Catalysis, Zürich CH 8092, Switzerland; ‡‡ Department of Environmental and Molecular Toxicology, Oregon State University, Corvallis Oregon 97331, United States

**Keywords:** PFASs, PFAS, health risks, health myths

## Abstract

A substantial body of evidence exists demonstrating that exposure to per- and polyfluoroalkyl substances (PFASs) poses a risk to human health. Data from epidemiological studies of those exposed occupationally or environmentally demonstrate adverse health risks, and these health effects are concordant with data from toxicological studies. Systematic reviews, conducted by agencies, organizations, and independent scientists that synthesize and integrate these data streams, have concluded that a range of health risks arise from PFAS exposure, including different types of cancer, especially kidney and testicular cancer, metabolic alterations such as increased liver enzymes and increased cholesterol, immune dysfunction such as reduced vaccination efficiency, reproductive and developmental outcomes such as low birth weight and reduced duration of breast feeding, and forms of endocrine disruption. Despite this, myths and misinformation surrounding these health risks slow efforts to protect public health from the hazards of PFAS exposure. This work addresses the most predominant of these myths and counters them with accumulated evidence from epidemiological and toxicological studies, demonstrating that exposure to PFASs poses a risk to human health.

## Graphical abstract

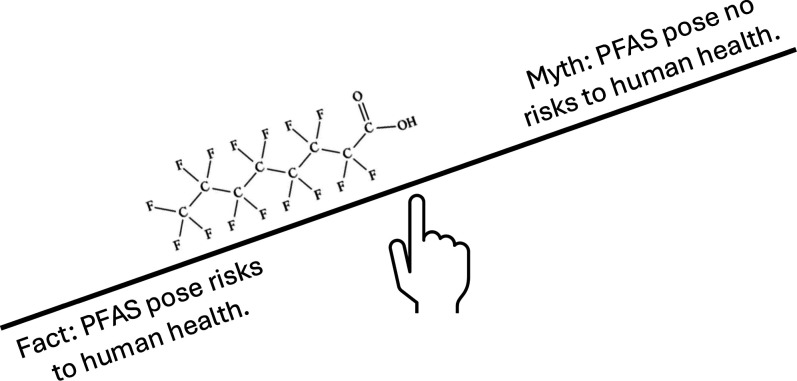

## 1 Introduction

Modern forms of environmental pollution from industrialization, intensive agriculture, and urbanization, including chemical pollution, contribute to human disease estimates of one in six deaths worldwide.^1^ Influences of chemical pollution are under-counted and difficult to quantify in totality. This is partly due to global dissemination of diverse chemical pollutants^1^ and because chemicals can have multiple physical, chemical, and toxicological effects and interact with each other and nonchemical stressors. A prominent class of such globally detected synthetic chemicals is per- and polyfluoroalkyl substances (PFASs).

PFASs have been incorporated into products and applications across multiple sectors, including consumer, industrial, manufacturing, pharmaceutical/medical, agricultural, and military products, due to convenient chemical characteristics such as stability, water- and oil-repellency, or surfactant properties.^2^ The ubiquity of PFASs recently led Dunn et al.^3^ to describe a “PFASphere” where PFASs used across different sectors all lead back to human exposures, which are virtually inescapable.

Of the thousands of PFAS structures identified,^4^ a relatively small number have been studied epidemiologically and/or toxicologically. Two PFASs, perfluorooctanoic acid (PFOA) and perfluorooctanesulfonic acid (PFOS), along with relatively small numbers of other PFASs, mostly those classified as perfluoroalkyl acids (PFAAs), form the basis for most health-related and mechanistic data. Thus, when health effects of PFASs are described, they likely concern PFOA, PFOS, and highly related substances of varying carbon-chain lengths such as homologues and precursors that often are referred to as “long-chain” or “legacy” PFASs. Studies of other PFASs, such as those termed “short-chain” or “alternative”, suggest that they are not without potential for biological activity, but evidence on hazards is still emerging.^5–8^

Several regulatory and health advisory agencies in North America and the European Union have reviewed epidemiological and toxicological data for some PFASs and determined that exposure to these PFASs increases the risk of certain diseases.^9–16^ Because not all organizations use aligned approaches for determining and communicating health risks, not all agree on the strength of the evidence for health risks associated with PFASs. These different agencies have identified^9–16^ the most common diseases linked to some PFASs, including.

1) different types of cancer, especially kidney and testicular cancer
2) metabolic alterations such as increased liver enzymes and increased cholesterol
3) immune dysfunction such as reduced vaccination efficiency
4) reproductive and developmental outcomes such as low birth weight and reduced duration of breast feeding
5) forms of endocrine disruption.

Despite this large body of evidence linked to the aforementioned chronic or developmental PFAS exposures, misleading language has led to the emergence of several enduring and unhelpful myths about PFAS health risks. Such myths obscure scientific understanding, complicate public health communications, and conflate uncertainty. This Perspective highlights some common PFAS health myths and provides evidence from scientific data streams that refute them.

## 2 Health Myths

### 2.1 MYTH 1: PFASs Cannot be Toxic because They are Chemically “Inert”

A common misconception is that because many PFASs are stable and nonreactive, i.e., chemically inert, they cannot produce toxicity in living organisms.^17^ Toxicity requires delivery of a chemical to its target, which involves absorption across cell membranes (or damage to membranes), distribution throughout the organism, and/or interactions with biological molecules.^18^ Studies of humans from different geographical locations across the globe demonstrate that many PFASs are detectable in human blood,^19^ which indicates that they can cross barriers to absorption designed to protect the body. Once in the blood, PFASs, like any chemical absorbed into the blood, can be transported to interact with different tissues and cells.^18^ Many PFASs share a resemblance with fatty acids,^20^ compounds essential for cell structure and energy storage. As a result, they can bind to fatty acid-associated and other proteins, including transport proteins (human serum albumin (HSA), liver fatty acid binding proteins, and organic acid transporters), and nuclear receptors (peroxisome proliferator activated receptors and hormone receptors) via hydrogen bonds.^21^ PFASs binding to these proteins can block essential chemicals from performing their normal functions or activate signals at the wrong place and time. When PFASs bind to HSA, a carrier protein for hormones and fatty acids, the transport of endogenous chemicals is reduced, leading to inappropriate or underpowered signals.^20^ When PFASs bind to the peroxisome proliferator activated receptor alpha (PPARα), a transcription factor that controls gene expression for energy homeostasis and inflammatory processes, altered metabolic processes and inappropriate inflammation may result.^22^

Many PFASs have been reported to bind to other proteins, affecting various functions across cell and tissue types, and some PFASs also can impact the integrity of cell and mitochondrial membranes, affecting their fluidity and subsequent cellular functions and survival.^7^ While PFAAs are chemically inert (although not all PFASs are chemically inert), interactions with these different biological matrices may lead to changes at the site of interaction or may induce downstream effects, all of which may alter physiology. Over time, these changes may increase the risk of people developing PFAS-linked disease. Thus, the PFAS toxicity is driven by these biological interactions. Even highly soluble, nonadsorptive PFAAs such as the ultrashort-chain trifluoroacetic acid (TFA) interact biologically, but their rapid elimination tends to make them less toxicologically potent than long-chain PFAAs.^23^ Environmental levels of these highly mobile PFAAs are increasing globally, raising concerns about long-term exposure despite their lower bioaccumulation.^22^

### 2.2 MYTH 2: The presence of PFASs in People Across the Globe is Evidence that PFASs are Not Harmful

While ubiquitous exposure and body burdens for many PFASs have been well documented through biomonitoring studies worldwide, adequately evaluating the health effects of widespread, slow-moving, and complex chemical exposure remains a global challenge. Evaluating impacts of chemicals with long half-lives – the case for many PFASs – that affect development or lead to effects from chronic exposures is more difficult than evaluating impacts of short-term, acute toxicity from exposure to high concentrations of environmental chemicals. It is well documented that chronic, noncommunicable disease incidence is increasing globally, and cardiovascular, respiratory, and metabolic disorders have all been linked to environmental chemicals.^24^ Often, insufficient data on exposure and environmental concentrations hamper the ability to make connections between exposures and chronic disease. Moreover, critical exposures or exposure windows may be far removed, temporally, from the development of the disease. These challenges in identifying long-term associations are compounded when a chemical selectively impacts a subset of the population, such as those with a specific genetic background, a certain age group, or lifestage, or when it acts in concert with other contaminants or stressors that are not as widely distributed. Additionally, combined exposures to PFASs are the rule rather than the exception, and their mixture toxicity is still being explored. Despite incomplete data and uncertainties and as discussed below, mounting evidence points to human health effects associated with many PFASs.

### 2.3 MYTH 3: Human health Effects from Exposure to PFASs in the Environment are Unknown or if They Exist, are Not Clinically Relevant

The scientific understanding of risks from exposure to potentially hazardous chemicals in the environment evolves from multiple lines of evidence across human populations, environmental occurrence in air, water, and soil paired with uptake by wildlife and plants used for human consumption, experimental animal species, in vitro, and in silico studies. These diverse study designs are synthesized and integrated to inform about human health impacts.^25^ The overall finding from these lines of evidence is that human health risks attributed to many PFASs have been identified through studies of occupationally and environmentally exposed human populations, and this is supported by extensive evidence from experimental animal model studies.

One of the largest epidemiological studies was conducted around Parkersburg, West Virginia (USA), a community exposed to PFOA in drinking water for decades due to a PFAS production facility. The C8 Science Panel identified probable links from PFOA exposures to clinically important diseases, including kidney and testicular cancer, elevated cholesterol, pregnancy-induced hypertension and preeclampsia, thyroid disease, and ulcerative colitis.^26^ Additional epidemiological studies have identified reduced responses to vaccines, liver and kidney damage, metabolic impairments, and adverse reproductive and developmental outcomes.^22^ These and additional epidemiological and toxicological data have been evaluated in systematic reviews by organizations such as the United States Environmental Protection Agency (US EPA) and Agency for Toxic Substances and Disease Registry (ATSDR), Health Canada, the European Food Safety Authority (EFSA), the US National Academies, and the International Agency for Research on Cancer (IARC) to independently determine that exposure to PFASs poses human health risks.^9–16^

“Clinical relevance” describes tangible or meaningful implications of a discovery in real-life situations.^27^ For many PFASs, evidence of liver damage, elevated cholesterol, and reduced responses to vaccines has been questioned as not being “clinically relevant” as some studies report median or average values within a normal clinical range. As an example, a reduced response to a vaccine does not typically produce clinical symptoms or disease in the immune system, but does indicate that the immune system is impaired.^28^ A lower population response to a vaccine, even within an accepted range around normal values, is evidence that some individuals within the population will have a response below the normal range.^28^ This lower vaccine response increases the risk for the population of developing infectious diseases, especially for those vulnerable, such as children and the elderly.^29^ “Clinical relevance” is, therefore, not an appropriate criterion for this effect. If there is clear evidence of higher population level rates of any type of infection, the entire population is at risk and ignoring it is contrary to protecting public health.^29^ Thus, using a measure of immune system function is an appropriate measure of immune system impairment.^29^

### 2.4 MYTH 4: Human Health Effects from Populations Highly Exposed to PFASs do Not Exist

One of the first studies of health effects of PFOA in humans was an occupational study reporting that 10 years of employment with elevated exposure to PFOA increased prostate cancer mortality by 3.3-fold compared to no employment in PFOA production.^30^ A follow-up study reported no associations between PFOA and risk of dying from any type of cancer but did report mild increases in hazard ratios of several types of cancers.^31^ A study of a different highly exposed occupational population reported an increased risk of deaths from kidney cancer with PFOA exposure.^32^ Other studies of highly exposed populations have reported increases in cholesterol, reproductive hormone levels, ulcerative colitis, and markers of liver and kidney damage with PFOA, PFOS, and/or other PFASs exposure.^12^ Ongoing work with firefighters, for example, with occupational exposures to PFASs demonstrates changes in molecular signals related to various diseases, including cancers.^33,34^ Various other studies of highly exposed populations have reported mixed findings. Explanations exist for mixed findings of such studies, especially occupational studies. One is the “healthy worker effect,” as people who work tend to be healthier than those who do not work and have potentially lower susceptibility to hazardous exposures.^35^ Another is intentional manipulation of study design, data, and/or comparators to reduce or eliminate the chance of finding positive outcomes.^17^ Published systematic reviews by regulatory and health advisory agencies contain excellent summaries of studies of highly exposed populations and their outcomes as well as evaluations of risk of bias for these studies.^9–16^

### 2.5 MYTH 5: Links Between Exposure to PFASs and Cancer in Humans are Weak and Due to Chance, Confounding, and/or Bias

Regulatory and health advisory agencies in North America and the European Union and a committee of the US National Academies of Sciences, Engineering, and Medicine (NASEM) have all reviewed cancer hazards of PFOA and PFOS and most report evidence of a positive relationship between PFAS exposure and cancer risks (the NASEM committee evaluated available data for seven different PFASs, including PFOA and PFOS).^9–16^ The IARC classified PFOA as carcinogenic to humans (Group 1) and PFOS as possibly carcinogenic to humans (Group 2B).^16^ Regarding cancer in humans, the IARC indicated that there was limited evidence of carcinogenicity for PFOA and inadequate evidence regarding carcinogenicity for PFOS. “Limited evidence” is “a causal interpretation of the positive association observed in the body of evidence on exposure to the agent and cancer is credible, but chance, bias, or confounding could not be ruled out with reasonable confidence.^16^” “Inadequate evidence” is “the available studies are of insufficient quality, consistency, or statistical precision to permit a conclusion to be drawn about the presence or the absence of a causal association between exposure and cancer, or no data on cancer in humans are available.^16^” Importantly, these definitions refer to the strength of the epidemiological evidence and not to the risk of cancer. The IARC noted that while more than three dozen studies evaluating cancer in humans were available for PFOA and PFOS, just under 10 studies examined risk for specific types of cancer, which is a very small number of studies across different cancers.^16^ While the epidemiological evidence for carcinogenicity of PFOA in humans was regarded as limited, PFOA was elevated to Group 1 based on experimental animal and mechanistic data. Data of carcinogenicity from experimental animals were sufficient: Exposure to PFOA caused an increase in the incidence of an appropriate combination of benign and malignant neoplasms in both sexes of a single species (rat) in one study that complied with Good Laboratory Practices.^16^ Additionally, the mechanistic evidence was strong: PFOA exposure induces epigenetic alterations and is immunosuppressive, which are key characteristics of carcinogens.^16^ PFOS was classified as a Group 2B carcinogen based on strong mechanistic evidence, i.e., PFOS, like PFOA, induces epigenetic alterations and is immunosuppressive.^16^ Thus, in the IARC assessment of PFOA and PFOS, toxicological and mechanistic data were supportive of epidemiological data.

The US EPA classified both PFOA and PFOS as “likely human carcinogens.^9,10^ ” Available data for both PFOA and PFOS demonstrated positive findings in animal experiments in more than one species, sex, strain, site, or exposure route, a rare animal tumor response in a single experiment assumed relevant to humans, and a positive tumor study strengthened by other lines of evidence.^9,10^ Data for PFOA were strengthened by a plausible association between human exposure and kidney and testicular cancers in the general population and in highly exposed populations.^9^ Data for PFOS demonstrated that although epidemiological evidence of associations between PFOS exposure and cancer were mixed across tumor types, available study findings supported a plausible correlation between PFOS exposure and carcinogenicity in humans.^10^

The NASEM committee found sufficient evidence of an association with increased risk of kidney cancer and limited or suggestive evidence of an association with increased risk of testicular cancer.^15^ “Sufficient evidence of an association” means that based on strong evidence, there is high confidence that there is an association between exposure to PFAS and the health outcome and it is unlikely that the association is due to chance or bias.^15^ “Limited or suggestive evidence of an association” means that based on limited evidence, there is moderate confidence that there is an association between exposure to PFASs and the health outcome and it is possible that the association is due to chance or bias.^15^ Health Canada noted that for PFASs other than PFOA and PFOS, risk of cancer remains inconsistent,^13^ and EFSA concluded that there was limited evidence for PFAS carcinogenicity based on data evaluated at the time (in 2018).^14^

While the IARC, US EPA, and the NASEM committee acknowledged that chance, confounding, or bias was possible, the overall body of evidence showed a carcinogenic effect. These classifications arose from a systematic review of evidence by independent scientists and had various levels of peer-review. Each of these entities also reached a conclusion independently of the others that PFOA and PFOS pose a carcinogenic hazard to humans.

### 2.6 MYTH 6: PFAS Health Effects in Experimental Rodent Models are Not Relevant to Humans Because Rodents are Exposed to Levels of PFASs Much Higher than Human Exposures, and Besides, Rodent Health Effects are Mediated by PPARα, which is Not Relevant to Humans

Rodent models of toxicity have long served as valuable tools for understanding chemical mechanisms of toxicity, biologic activity, and potential hazards to human health, which are used to assess chemical risks to humans across many different regulatory and health agencies, such as those previously discussed. These rodent models continue to provide critical data for understanding the wide range of PFAS-associated health effects.^22^ Toxicity studies in experimental rodent models can be conducted in myriad ways, but harmonized guidelines, such as those described by the Organisation for Economic Co-operation and Development (OECD) and the US EPA are available and followed in many assessments. An OECD test guideline for a 28-day repeated dose oral toxicity study in rodents (OECD Test Guideline No. 407) indicates that “the highest dose level should be chosen with the aim of inducing toxic effects but not death or severe suffering.^36^” In other words, the highest dose level administered should produce toxicity. This guideline does not address “human relevant doses” or “human equivalent exposures.” The US EPA guideline for determination of combined chronic toxicity/carcinogenicity (OPPTS 870.4300) states that “the highest dose level in rodents should elicit signs of toxicity without substantially altering the normal life span due to effects other than tumors.^37^” Thus, when evaluating toxicity of various substances in experimental rodent models, the goal is not to reproduce human exposures but to elucidate toxicological outcomes at doses that produce toxicity in the experimental rodent model.

For many substances, assessing the risk of human health effects from exposure often requires extrapolation of experimental rodent model data to humans. The extrapolations can over- or underestimate risk and generally include various uncertainty and adjustment factors to account for differences between rodents and humans, heterogeneity in the human population, and adequacy of the evidence base.^38^ It is not a requirement that experimental rodent model data reflect human doses, as the human exposure scenario is often difficult to capture in a controlled experiment.

For PFASs, experimental rodent model data are copious, and the database from epidemiological studies in humans is substantial for a variety of health outcomes. A key point here is that concordance between observations in experimental rodent model data and epidemiological findings is high; many of the effects observed in laboratory studies of experimental rodent models also have been observed in studies of PFAS-exposed humans. Thus, findings in experimental rodent models substantiate epidemiological findings, and vice versa.

One molecular mechanism by which some PFASs are thought to produce toxicity is through activation of PPARα, which plays a major role in metabolic regulation of living organisms.^39^ Briefly, when bound by a PFAS or an endogenous chemical, PPARα binds to specific DNA regions to affect expression of target genes.^40^ Gene expression profiling has revealed hundreds of PPARα-target genes that can be differentially affected, leading to myriad metabolic functions in multiple organs.^39^ In experimental rodent models treated with synthetic agents that bind PPARα, liver enlargement, proliferation of subcellular organelles known as peroxisomes, and eventual development of liver tumors are observed; these specific changes have not been observed in humans.^40^ However, this is not proof that these effects or the involvement of PPARα is not relevant to humans. What is decisive is that studies comparing rodent and human PPARα responsivity to PFASs in vitro report that, depending on the PFASs applied, human PPARα appears to be as sensitive or even more sensitive than rodent PPARα.^41–43^ Nevertheless, dogma has emerged, suggesting that a PPARα mechanism of action for “PFAS toxicity” is irrelevant to humans. There is some evidence that humans are unlikely to develop liver tumors from PPARα activation by PFASs,^44^ but other types of toxicity, including elevated cholesterol and immunotoxicity, may very well arise from PPARα activation by PFASs in humans.^45^ Thus, liver tumors arising from PPARα activation by PFASs are the only health effect in experimental rodent models that is thought to be unlikely to occur in humans. Other health effects arising from PPARα-dependent and PPARα-independent mechanisms of action in rodents are completely plausible for humans.

## 3 What Really Matters

Humans are already being exposed to multiple chemicals in various environmental media, including drinking water^46^ and food.^19^ Due to the poor reversibility of PFAS pollution and hence exposure, risks will likely increase until action is taken to reduce PFASs as a class rather than one PFAS at a time^47^ as a preventative approach to avoid further harm.^48^ ^,^ ^49^

The longer that misinformation on the health risks of PFASs acts as a means to delay action on phasing out or otherwise restricting their use, the longer communities remain exposed, perpetuating these potential harms. PFOA, PFOS, and many other PFASs are hazards to human health. The ubiquity of PFASs across myriad sectors all lead back to human exposures and exposures lead to health risks. That is no myth.
