# Correction to “PFAS Toxicity: What’s True, What’s Not, and What Really Matters”

**DOI:** 10.1021/acs.est.6c11134

**Published:** 2026-08-07

**Authors:** Jamie C. DeWitt, Ian T. Cousins, Gretta Goldenman, Dorte Herzke, Rainer Lohmann, Mark Miller, Carla A. Ng, Martin Scheringer, Xenia Trier, Zhanyun Wang

An error occurred with in-text citations and references. The affected paragraphs with updated citations and the corrected references are shown below.

## Corrected Paragraphs (note that corrections are bolded)

Many PFASs have been reported to bind to other proteins, affecting various functions across cell and tissue types, and some PFASs also can impact the integrity of cell and mitochondrial membranes, affecting their fluidity and subsequent cellular functions and survival.^7^ While PFAAs are chemically inert (although not all PFASs are chemically inert), interactions with these different biological matrices may lead to changes at the site of interaction or may induce downstream effects, all of which may alter physiology. Over time, these changes may increase the risk of people developing PFAS-linked disease. Thus, the PFAS toxicity is driven by these biological interactions. Even highly soluble, nonadsorptive PFAAs such as the ultrashort-chain trifluoroacetic acid (TFA) interact biologically, but their rapid elimination tends to make them less toxicologically potent than long-chain PFAAs.^23^ Environmental levels of these highly mobile PFAAs are increasing globally, raising concerns about long-term exposure despite their lower bioaccumulation.^**24**^

While ubiquitous exposure and body burdens for many PFASs have been well documented through biomonitoring studies worldwide, adequately evaluating the health effects of widespread, slow-moving, and complex chemical exposure remains a global challenge. Evaluating impacts of chemicals with long half-lives – the case for many PFASs – that affect development or lead to effects from chronic exposures is more difficult than evaluating impacts of short-term, acute toxicity from exposure to high concentrations of environmental chemicals. It is well documented that chronic, noncommunicable disease incidence is increasing globally, and cardiovascular, respiratory, and metabolic disorders have all been linked to environmental chemicals.^**22**^ Often, insufficient data on exposure and environ-mental concentrations hamper the ability to make connections between exposures and chronic disease. Moreover, critical exposures or exposure windows may be far removed, temporally, from the development of the disease. These challenges in identifying long-term associations are com-pounded when a chemical selectively impacts a subset of the population, such as those with a specific genetic background, a certain age group, or lifestage, or when it acts in concert with other contaminants or stressors that are not as widely distributed. Additionally, combined exposures to PFASs are the rule rather than the exception, and their mixture toxicity is still being explored. Despite incomplete data and uncertainties and as discussed below, mounting evidence points to human health effects associated with many PFASs.

One of the largest epidemiological studies was conducted around Parkersburg, West Virginia (USA), a community exposed to PFOA in drinking water for decades due to a PFAS production facility. The C8 Science Panel identified probable links from PFOA exposures to clinically important diseases, including kidney and testicular cancer, elevated cholesterol, pregnancy-induced hypertension and preeclampsia, thyroid disease, and ulcerative colitis.[Fn ref26] Additional epidemiological studies have identified reduced responses to vaccines, liver and kidney damage, metabolic impairments, and adverse reproductive and developmental outcomes.[Fn ref27] These and additional epidemiological and toxicological data have been evaluated in systematic reviews by organizations such as the United States Environmental Protection Agency (US EPA) and Agency for Toxic Substances and Disease Registry (ATSDR), Health Canada, the European Food Safety Authority (EFSA), the US National Academies, and the International Agency for Research on Cancer (IARC) to independently determine that exposure to PFASs poses human health risks.^9–16^

“Clinical relevance” describes tangible or meaningful implications of a discovery in real-life situations.^**28**^ For many PFASs, evidence of liver damage, elevated cholesterol, and reduced responses to vaccines has been questioned as not being “clinically relevant” as some studies report median or average values within a normal clinical range. As an example, a reduced response to a vaccine does not typically produce clinical symptoms or disease in the immune system, but does indicate that the immune system is impaired.^**29**^ A lower population response to a vaccine, even within an accepted range around normal values, is evidence that some individuals within the population will have a response below the normal range.^**29**^ This lower vaccine response increases the risk for the population of developing infectious diseases, especially for those vulnerable, such as children and the elderly.^**30**^ “Clinical relevance” is, therefore, not an appropriate criterion for this effect. If there is clear evidence of higher population level rates of any type of infection, the entire population is at risk and ignoring it is contrary to protecting public health.^**30**^ Thus, using a measure of immune system function is an appropriate measure of immune system impairment.^**30**^

One of the first studies of health effects of PFOA in humans was an occupational study reporting that 10 years of employment with elevated exposure to PFOA increased prostate cancer mortality by 3.3-fold compared to no employment in PFOA production.^**31**^ A follow-up study reported no associations between PFOA and risk of dying from any type of cancer but did report mild increases in hazard ratios of several types of cancers.^**32**^ A study of a different highly exposed occupational population reported an increased risk of deaths from kidney cancer with PFOA exposure.^**33**^ Other studies of highly exposed populations have reported increases in cholesterol, reproductive hormone levels, ulcerative colitis, and markers of liver and kidney damage with PFOA, PFOS, and/or other PFASs exposure.^12^ Ongoing work with fire-fighters, for example, with occupational exposures to PFASs demonstrates changes in molecular signals related to various diseases, including cancers.^**34**,**35**^ Various other studies of highly exposed populations have reported mixed findings. Explanations exist for mixed findings of such studies, especially occupational studies. One is the “healthy worker effect,” as people who work tend to be healthier than those who do not work and have potentially lower susceptibility to hazardous exposures.^**36**^ Another is intentional manipulation of study design, data, and/or comparators to reduce or eliminate the chance of finding positive outcomes.^17^ Published systematic reviews by regulatory and health advisory agencies contain excellent summaries of studies of highly exposed populations and their outcomes as well as evaluations of risk of bias for these studies.^9–16^

Rodent models of toxicity have long served as valuable tools for understanding chemical mechanisms of toxicity, biologic activity, and potential hazards to human health, which are used to assess chemical risks to humans across many different regulatory and health agencies, such as those previously discussed. These rodent models continue to provide critical data for understanding the wide range of PFAS-associated health effects.^22^ Toxicity studies in experimental rodent models can be conducted in myriad ways, but harmonized guidelines, such as those described by the Organisation for Economic Co-operation and Development (OECD) and the US EPA are available and followed in many assessments. An OECD test guideline for a 28-day repeated dose oral toxicity study in rodents (OECD Test Guideline No. 407) indicates that “the highest dose level should be chosen with the aim of inducing toxic effects but not death or severe suffering.^**37**^” In other words, the highest dose level administered should produce toxicity. This guideline does not address “human relevant doses” or “human equivalent exposures.” The US EPA guideline for determination of combined chronic toxicity/carcinogenicity (OPPTS 870.4300) states that “the highest dose level in rodents should elicit signs of toxicity without substantially altering the normal life span due to effects other than tumors.^**38**^” Thus, when evaluating toxicity of various substances in experimental rodent models, the goal is not to reproduce human exposures but to elucidate toxicological outcomes at doses that produce toxicity in the experimental rodent model.

For many substances, assessing the risk of human health effects from exposure often requires extrapolation of experimental rodent model data to humans. The extrapolations can over- or underestimate risk and generally include various uncertainty and adjustment factors to account for differences between rodents and humans, heterogeneity in the human population, and adequacy of the evidence base.^**39**^ It is not a requirement that experimental rodent model data reflect human doses, as the human exposure scenario is often difficult to capture in a controlled experiment.

One molecular mechanism by which some PFASs are thought to produce toxicity is through activation of PPARα, which plays a major role in metabolic regulation of living organisms.^**40**^ Briefly, when bound by a PFAS or an endogenous chemical, PPARα binds to specific DNA regions to affect expression of target genes.^40^ Gene expression profiling has revealed hundreds of PPARα-target genes that can be differentially affected, leading to myriad metabolic functions in multiple organs.^**40**^ In experimental rodent models treated with synthetic agents that bind PPARα, liver enlargement, proliferation of subcellular organelles known as peroxisomes, and eventual development of liver tumors are observed; these specific changes have not been observed in humans.^**41**^ However, this is not proof that these effects or the involvement of PPARα is not relevant to humans. What is decisive is that studies comparing rodent and human PPARα responsivity to PFASs in vitro report that, depending on the PFASs applied, human PPARα appears to be as sensitive or even more sensitive than rodent PPARα.^**42–44**^ Nevertheless, dogma has emerged, suggesting that a PPARα mechanism of action for “PFAS toxicity” is irrelevant to humans. There is some evidence that humans are unlikely to develop liver tumors from PPARα activation by PFASs,^**45**^ but other types of toxicity, including elevated cholesterol and immunotoxicity, may very well arise from PPARα activation by PFASs in humans.^**46**^ Thus, liver tumors arising from PPARα activation by PFASs are the only health effect in experimental rodent models that is thought to be unlikely to occur in humans. Other health effects arising from PPARα-dependent and PPARα-independent mechanisms of action in rodents are completely plausible for humans.

Humans are already being exposed to multiple chemicals in various environmental media, including drinking water^**47**^ and food.^19^ Due to the poor reversibility of PFAS pollution and hence exposure, risks will likely increase until action is taken to reduce PFASs as a class rather than one PFAS at a time^**48**^ as a preventative approach to avoid further harm.^48,49^

## Footnotes

26 C8 Probable Link Reports. http://www.c8sciencepanel.org/prob_link.html (accessed 2026-01-07).

27 National Academies of Sciences, Engineering, and Medicine. Building confidence in new evidence streams for human health risk assessment: Lessons learned from laboratory mammalian toxicity tests. The National Academies Press: Washington, DC, 2023.

